# A Microscale Platform for the Comprehensive Analysis of Bacterial Translation Initiation

**DOI:** 10.3390/ijms27114953

**Published:** 2026-05-29

**Authors:** Daria S. Vinogradova, Pavel S. Kasatsky, Zoya A. Spiridonova, Sebastian Leyva, Ana Sanchez-Castro, Katherin Peñaranda, Victor Zegarra, Pablo Soriano, Alena Paleskava, Pohl Milon, Andrey L. Konevega

**Affiliations:** 1 Petersburg Nuclear Physics Institute Named by B.P. Konstantinov of National Research Centre “Kurchatov Institute”, Gatchina 188300, Russiaspiridonova_za@pnpi.nrcki.ru (Z.A.S.); polesskova_ev@pnpi.nrcki.ru (A.P.); konevega_al@pnpi.nrcki.ru (A.L.K.); 2 Biomolecules Laboratory, Faculty of Health Sciences, Universidad Peruana de Ciencias Aplicadas (UPC), Lima 15023, Perukatherin.penaranda@upc.pe (K.P.); pmilon@upc.pe (P.M.); 3 School of Biology, Faculty of Health Sciences, Universidad Peruana de Ciencias Aplicadas (UPC), Lima 15023, Peru; 4 Max Planck Institute for Biological Intelligence, 82152 Martinsried, Germany; victor.zegarra@bi.mpg.de; 5 Institute of Biomedical Systems and Biotechnologies, Peter the Great St. Petersburg Polytechnic University, Saint Petersburg 195251, Russia; 6 National Research Centre “Kurchatov Institute”, Moscow 123182, Russia

**Keywords:** prokaryotic translation initiation, ribosome assembly, protein synthesis inhibitors, antibiotics, antimicrobial peptides, fluorescence, microscale thermophoresis, nano differential scanning fluorimetry

## Abstract

In prokaryotes, translation initiation orchestrates protein synthesis through a network of dynamic interactions among the ribosome, mRNA, initiator tRNA^fMet^, and initiation factors (IFs). Traditional approaches that rely on radioactive labeling or surface immobilization are hindered by inherent safety risks and methodological constraints. We present a fluorescence-based analytical platform that integrates microscale thermophoresis (MST) as a unified, multiparametric toolkit for comprehensive interrogation of bacterial translation initiation at the molecular level. By systematically applying MST to a panel of fluorescently labeled components—initiator tRNA^fMet^, mRNAs, and initiation factors—we quantify assembly pathways and equilibria as initiation progresses from simple bimolecular interactions to higher-order, multicomponent complexes. To broaden the fluorescence toolbox for ribosomal studies, we developed a robust BODIPY-labeling protocol for 70S ribosomes and confirmed preservation of structural integrity and function by nano differential scanning fluorimetry, stopped-flow kinetic assays, and peptide-synthesis activity tests. Our microscale fluorescent system facilitates probing initiation at a variety of steps, since the role of magnesium ions and initiation factors upon 30S initiation complex formation. The same platform can be applied to investigate the effects of different compounds on translation initiation, as demonstrated for a number of antibiotics, aptamers, and antimicrobial peptides. Using this approach, we determined the antibiotic streptomycin dissociation constant for both 30S and 70S ribosomes, which proved identical at 0.3 ± 0.1 μM, and demonstrated the effect of the antimicrobial peptide rumicidin-1 on translation initiation. Offering a cost-effective and high-sensitivity alternative to conventional methods, this approach advances mechanistic understanding of prokaryotic translation and provides a versatile framework for the discovery of novel protein synthesis inhibitors.

## 1. Introduction

**Figure 1:**
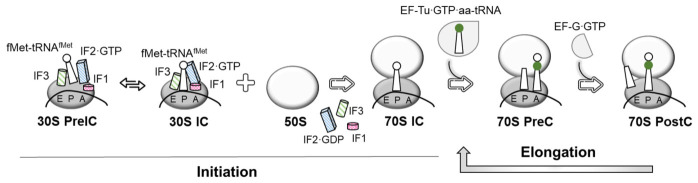
Schematic representation of initiation and elongation of bacterial translation cycles. Initiation starts on the binding of the initiator fMet-tRNA^fMet^ and mRNA to the small (30S) ribosomal subunit in the presence of initiation factors (IF1, IF2, IF3), forming a transient 30S pre-initiation complex (30S pre-IC). Upon correct codon–anticodon pairing, the equilibrium shifts toward the assembly of a stable 30S initiation complex (30S IC). Subsequent joining of the large (50S) subunit and release of initiation factors yield the mature 70S initiation complex (70S IC), competent for polypeptide synthesis. The elongation stage, which follows initiation, consists of decoding, peptide bond formation, and translocation steps. The aminoacyl-tRNA (aa-tRNA), as part of a ternary complex with the elongation factor EF-Tu and GTP, is recruited to the ribosomal A site. Following successful codon–anticodon recognition between the aa-tRNA and mRNA, GTP hydrolysis, and dissociation of EF-Tu from the ribosome, peptide bond formation occurs in the peptidyl transferase center of the 50S ribosomal subunit. Following the subsequent translocation of mRNA and tRNA relative to the 30S subunit, catalyzed by the GTPase elongation factor EF-G, the next mRNA codon becomes exposed in the ribosomal A site, ready to accept the cognate aminoacyl-tRNA. The deacylated tRNA leaves the ribosome through the E site. The process ends in the termination stage, where termination factors RF1 or RF2 recognize the stop codon on the mRNA and catalyze the release of the polypeptide chain from the P-site tRNA.

Messenger RNA (mRNA) translation constitutes a fundamental biological process that decodes genetic information into functional proteins. While the overall mechanism is conserved among archaea, bacteria, and eukaryotes, the initiation phase exhibits substantial divergence across these domains [1]. In bacteria, the rate-limiting step of translation initiation entails coordinated interactions among the 30S ribosomal subunit, mRNA, initiator fMet-tRNA^fMet^ (tRNA^fMet^), and the three initiation factors IF1, IF2, and IF3 [2,3,4] (Figure 1). Within the canonical pathway, the stable 30S initiation complex (IC) forms through codon–anticodon pairing between mRNA and tRNA^fMet^, followed by 50S subunit joining to yield the mature 70S IC competent for polypeptide synthesis [4,5]. This highly coordinated sequence of events governs both the rate and fidelity of translation. The initiation machinery integrates a network of intra- and intermolecular interactions that shape the cellular proteome [4,6]. Owing to its central role and molecular complexity, the bacterial initiation pathway represents a principal target for diverse translation inhibitors [2,7,8].

Differences in order, kinetics, and affinities of ligands’ interaction with the ribosome reveal the elaborate structure of the bacterial translation initiation process [5,9,10,11,12]. Conventional in vitro approaches rely on radioactively labeled ligands. It requires stringent safety procedures and entails substantial disposal costs. Fluorescence-based techniques provide a safer and more versatile alternative for investigating the biophysical and biochemical properties of translation [13,14,15,16]. Here, we go beyond simply applying fluorescence methods: we developed and integrated microscale thermophoresis (MST) into a unified, multiparametric platform that enables systematic, low-consumption measurement of binding equilibria, kinetics, and cooperative assembly across multiple initiation components and steps. This comprehensive MST toolkit not only removes the need for radioactivity but also permits parallel, quantitative interrogation of how magnesium concentration, individual initiation factors, and mRNA sequence influence 30S and 70S complex formation. One of the earliest applications of MST in translation research examined the stringent response in *Escherichia coli* (*E. coli*) by reconstituting the reaction with all essential components, including ribosomes, mRNA, initiator tRNA^fMet^, and initiation factors [15].

**Figure 2:**
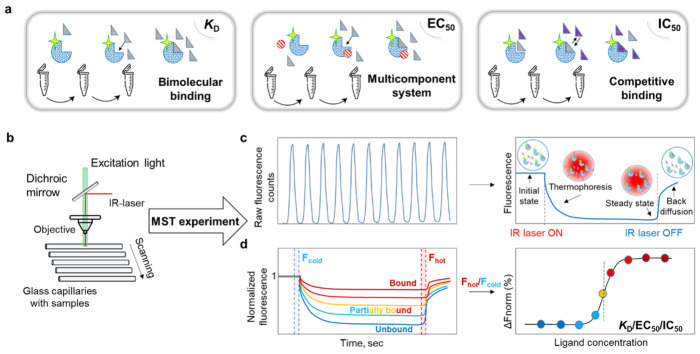
Microscale thermophoresis (MST) technology. (**a**) Schematic representation of an MST assay used to analyze bimolecular and multicomponent interactions. In a typical titration experiment, a fluorescently labeled reporter molecule (the yellow star symbolizes the fluorophore) is kept at a constant concentration, while the binding partner (ligand) is serially titrated. (**b**) Reaction samples are loaded into glass capillaries for analysis. (**c**) The absence of sample aggregation and pipetting errors is verified by monitoring the initial fluorescence signal. Each measurement follows this sequence: recording the initial fluorescence signal (“initial state”), activation of the infrared (IR) laser to induce thermal diffusion (“thermophoresis”) until equilibrium (“steady state”) is reached, and detection of the reverse diffusion process after the IR laser is switched off (“back diffusion”). (**d**) Representative MST time traces illustrating ligand binding. The assay monitors one fluorescently labeled ligand at a constant concentration while titrating the unlabeled ligand. Bound (red), partially bound (light red, orange, light blue), and unbound (blue) molecular states display distinct thermophoretic behaviors upon IR heating. The MST response is expressed as the normalized fluorescence ratio during and before heating (F_hot_/F_cold_). The MST signal correlates with the fraction of bound molecules, enabling the determination of ligand affinities and calculation of dissociation constants (*K*_D_), half-maximal effective concentrations (EC_50_), and half-maximal inhibitory concentrations (IC_50_).

Titration experiments using MST enable the determination of binding affinities across a wide range of dissociation constants (10^−12^–10^−3^ M, *K*_D_) and the quantification of inhibition (half-maximal inhibitory concentration, IC_50_) or activation (half-maximal effective concentration, EC_50_) with minimal sample volumes (<5 µL) (Figure 2a). Fluorescence from ligands—through intrinsic tryptophan residues or covalently attached fluorophores—allows monitoring molecular movement within glass capillaries. An infrared (IR) laser (1480 nm) locally heats approximately 2 nL of sample, generating a temperature gradient of 1–6 K (Figure 2b). The initial fluorescence measurement serves as a quality control step for detecting aggregation or pipetting inconsistencies. Upon IR laser activation, molecules undergo directed thermal diffusion until an equilibrium is reached; after the laser is switched off, back-diffusion occurs (Figure 2c). Thermophoretic mobility variations reflect binding-induced changes in molecular conformation, charge, size, or hydration shell. Measurements are performed without additional sample immobilization, thereby preserving near-native experimental conditions and highlighting the method’s equilibrium nature [17,18,19]. The thermophoretic curve reflects fluorescence changes as molecules accumulate or spread away from the observation point during heating cycles (Figure 2d).

We provide detailed quantitative insights into the fundamental steps of bacterial translation initiation using a diverse set of fluorescent reporter ligands. This microscale thermophoresis approach delivers precise binding affinity measurements while offering a cost-effective, accessible platform for investigating core biochemical principles of the process. Notably, the versatility of this approach extends to other complex molecular assemblies, particularly those involving components that are challenging to produce or obtainable only in limited quantities.

## 2. Results

### 2.1. A Novel Method for BODIPY Labeling of Ribosomes as Reporters

**Figure 3:**
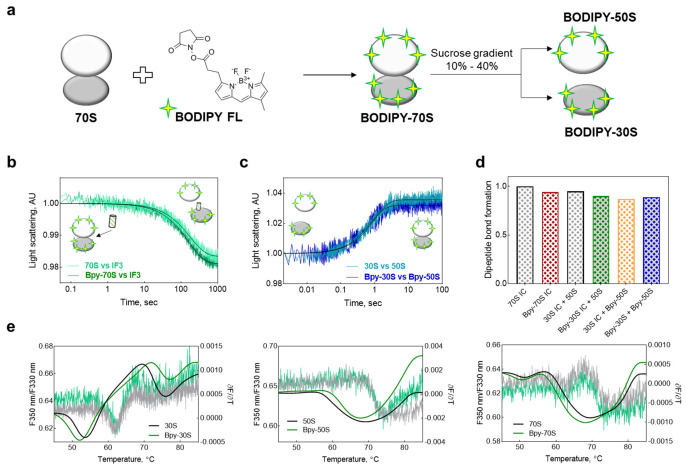
BODIPY fluorescent labeling of ribosomes. (**a**) Labeling scheme. After dissociation of BODIPY-70S ribosomes, the resulting BODIPY-labeled 30S and BODIPY-50S subunits were separated using a 10–40% sucrose gradient. (**b**) Light-scattering analysis of BODIPY-70S ribosome dissociation kinetics in the presence of initiation factor IF3, and (**c**) re-association of BODIPY-30S and BODIPY-50S subunits compared with native ribosomes. (**d**) Comparison of dipeptide synthesis efficiency using native and BODIPY-labeled 70S, 30S, and 50S ribosomes. (**e**) Melting curves of native and fluorescently labeled 30S, 50S, and 70S ribosomes measured by nanodifferential scanning fluorimetry (nanoDSF). The left axis shows the fluorescence ratio F350 nm/F330 nm, which reports on the intrinsic aromatic amino acid fluorescence change during thermal unfolding, with the largest contribution coming from tryptophans. The right axis shows the first derivative of this ratio with respect to temperature (∂F/∂T), which highlights the inflection point of the melting transition and is used to determine the melting temperature (Tm). The melting temperatures (Tm) were: 30S—53.6 °C, 69.2 °C; Bpy-30S—52.1 °C, 71.8 °C; 50S—70.1 °C; Bpy-50S—67.9 °C; 70S—51.3, 69.5; Bpy-70S—56.4 °C, 69.5 °C.

Visualizing ligand interactions on the ribosome is crucial for understanding translation; however, modifications such as fluorescent labeling must be carefully controlled to avoid disrupting molecule function. Methods for labeling ribosomes include modification of purified proteins or RNA followed by reconstitution of subunits [20,21], the use of reporter oligonucleotides for structural studies [22,23], the use of site-directed mutagenesis to attach dyes via cysteines [24,25], or labeling directly at lysine amino acid residues [3]. We introduce a fast and simple method for fluorescently labeling lysine residues in *E. coli* 70S ribosomes with BODIPY (Bpy) dye. Considering the large number of lysine residues in ribosome proteins, we optimized the necessary dye excess to preserve ribosome functional activity while maximizing the sensitivity of fluorescence signal changes during analysis. Importantly, to obtain Bpy-subunits, it is essential to label the entire 70S ribosomes prior to subunit splitting, since labeling the 30S and 50S subunits directly alters the intersubunit surface (Figure 3a), potentially hampering 70S formation. We assessed the labeling efficiency using SDS-PAGE (Figure S1a).

#### 2.1.1. Functional Activity of BODIPY-Ribosomes

The association of 30S and 50S subunits into the 70S ribosome, as well as the reverse process of dissociation into subunits, is critically important for maintaining a pool of functional ribosomes by regulating the balance between monomers and dimers [26,27]. We measured the kinetics of both reactions by the stopped-flow technique, monitoring the light-scattering signal change. The association rates of native and fluorescently labeled 30S and 50S subunits were very close, yielding values of 1.01 ± 0.02 s^−1^ and 1.33 ± 0.04 s^−1^, respectively (Figure 3c). The kinetics of 70S dissociation into subunits were measured in the presence of IF3 to exclude the re-association step [28,29,30,31,32]. The dissociation rates of native ((5.6 ± 0.1) × 10^−3^ s^−1^) and Bpy-70S ((6.2 ± 0.1) × 10^−3^ s^−1^) ribosomes were also virtually indistinguishable (Figure 3b). Importantly, the amplitudes of all the corresponding light-scattering signals were essentially identical, highlighting the equal amount of the functional ribosomes. Thus, fluorescent labeling of ribosomes had no impact on the structural integrity of the intersubunit interface and the dynamics of the ribosome assembly—critical hallmarks of translation efficiency.

Although the fluorescent dye uniformly localizes to ribosomal subunit surfaces without making significant contributions to the functionally active centers, we analyzed signal variations under translation initiation kinetics conditions. We monitored the formation of the 30S IC and 70S IC by recording changes in the fluorescence of labeled ribosomes (Figure S1b). As expected, assembly of the 30S initiation complex elicited no noticeable fluorescence signal shift, as ligand binding occurs in the intersubunit interface, the unlabeled region. Use of Bpy-70S or Bpy-30S and Bpy-50S ribosomes resulted in a marked increase in the fluorescence signal upon 70S initiation complex formation, with observed rates of 0.075 ± 0.004 min^−1^ and 0.049 ± 0.006 min^−1^ (Figure S1b), respectively. To confirm the functionality of the fluorescent initiation complexes, we tested their ability to participate in the peptidyl transferase reaction (Figure 3d). The use of Bpy-labeled 70S ribosomes, as well as isolated Bpy-30S and Bpy-50S subunits, supported efficient peptide bond formation comparable to that of native ribosomes.

#### 2.1.2. Probing Ribosomal Stability with Nano Differential Scanning Fluorimetry

Differential scanning calorimetry (DSC) and nano differential scanning fluorimetry (nanoDSF) are classical approaches for assessing the conformational stability of protein preparations [33,34,35,36]. We applied nanoDSF technology to monitor the preservation of conformational stability of native and fluorescently labeled 70S ribosomes, as well as in their 30S and 50S subunits (Figure 3e). Interestingly, the 30S subunit and 70S ribosome showed lower thermal stability (melting temperatures—Tm—53.6 °C and 51.3 °C, respectively) than the 50S subunit, which remained stable up to 70.1 °C under the conditions of the experiment. The higher stability of the large ribosomal subunit is most likely based on the high content of rigidly ordered and densely packed rRNA, forming, among other things, the evolutionarily most ancient parts of the ribosome, such as the peptidyl transferase center and the nascent peptide exit tunnel. The melting profiles of fluorescently labeled ribosomes were indistinguishable from those of their native counterparts (Figure 3e), confirming that labeling did not disrupt ribosomal stability. To more thoroughly examine the impact of the performed modification on ribosome conformational stability, we conducted nanoDSF analysis under varying ionic conditions, which play a decisive role in ribosomal structural and functional organization [37,38,39,40,41,42,43]. Among the major contributors, magnesium (Mg^2+^) and potassium (K^+^) ions are particularly essential to maintain the stability and translational competence of the 70S ribosome. Mg^2+^ primarily governs the structural integrity of the ribosome, whereas K^+^ ions influence both stability and translational dynamics [42,44,45,46,47,48,49,50,51] (Figure S1c). Melting curves of native and BODIPY-labeled 70S ribosomes, as well as 30S and 50S ribosomal subunits, remained identical across varying ionic conditions. Only the 50S subunit exhibited reduced sensitivity to Mg^2+^ concentration changes, which did not affect its activity in our conducted control analyses. A more detailed analysis of conformational stability dependence for native and modified ribosomes across varying ionic conditions is available in the Supplementary Materials.

### 2.2. Bimolecular Ligand Interactions in Translation Initiation Probed by MST

Assembly of the bacterial translation initiation complex proceeds through multiple sequential stages, requiring the presence of all ligands for optimal efficiency. Individual components, including IFs, tRNA^fMet^, and mRNA, form bimolecular complexes with the 30S ribosomal subunit with varying affinities [4,11,52,53,54]. We initiated MST studies of translation initiation through step-wise analysis. Initially, we examined bimolecular interaction affinities by determining dissociation constant (*K*_D_) values of ligands. We quantified the binding affinities of the IF1, IF2, IF3, mRNA, and tRNA^fMet^ to the 30S subunit, employing each as a fluorescent reporter in MST assays.

**Figure 4:**
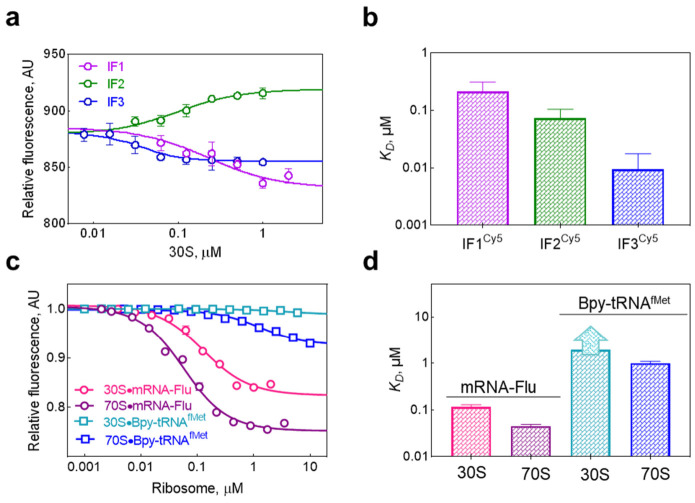
Determination of dissociation constants (*K*_D_) using different fluorescently labeled ligands as reporters. (**a**) Binding curves of IF1^Cy5^, IF2^Cy5^, and IF3^Cy5^ to the 30S ribosomal subunit. (**b**) The *K*_D_ values of IF1^Cy5^•30S (magenta), IF2^Cy5^•30S (green), and IF3^Cy5^•30S (blue) complexes. (**c**) Binding curves of mRNA-Flu or Bpy-tRNA^fMet^ to the 30S or 70S ribosomes. (**d**) The *K*_D_ values of mRNA-Flu•30S (magenta), mRNA-Flu•70S (violet), Bpy-tRNA^fMet^•30S (light blue), Bpy-tRNA^fMet^•70S (blue) complexes. Error bars represent standard deviations from three independent measurements. An upward arrow indicates lower bounds for *K*_D_ when the curve did not reach saturation (actual *K*_D_ is ≥the indicated value).

IF1, IF2, and IF3 were site-specifically fluorescently labeled with Cy5 via maleimide chemistry, targeting solvent-accessible cysteine residues to yield IF1^Cy5^, IF2^Cy5^, and IF3^Cy5^ (Figure S2a,b). The measured dissociation constants were 214 ± 98 nM for IF1, 73 ± 32 nM for IF2, and 9 ± 8 nM for IF3 in excellent agreement with values obtained previously by pre-steady-state kinetics (Figure 4a,b and Figure S2c) [10].

Although mRNA and tRNA^fMet^ can bind the 30S subunit independently, the 30S•mRNA complex exhibits substantially greater stability than the 30S•tRNA^fMet^ complex [55,56,57,58]. 30S•mRNA association is mediated by complementary base pairing between the anti-Shine–Dalgarno (anti-SD) sequence in 16S rRNA and the Shine–Dalgarno (SD) sequence in mRNA, positioning the mRNA within a dedicated channel on the subunit surface [4]. In contrast, formation of the 30S•tRNA^fMet^ bimolecular complex is less stable due to the requirement for the presence of IF2, which is responsible for recruiting tRNA^fMet^ to the P site of the ribosome [11,59].

We quantified the bimolecular interactions of mRNA and tRNA^fMet^ with 30S and 70S ribosomes using MST with fluorescein-labeled mRNA (mRNA-Flu) or BODIPY-labeled tRNA^fMet^ (Bpy-tRNA^fMet^) as the reporters (Figure 4c,d and Figure S2d). mRNA-Flu exhibited high-affinity binding to 30S and 70S, with dissociation constants (*K*_D_) of 115 ± 14 nM and 45 ± 4 nM respectively. Bpy-tRNA^fMet^ bound efficiently to 70S ribosomes (*K*_D_ = 1.0 ± 0.1 μM), but showed markedly weaker affinity for 30S alone (the *K*_D_ value could not be determined).

### 2.3. MST Measurements of Initiation Complex Formation Efficiency

Bacterial translation initiation represents a multifaceted process that can proceed via distinct pathways involving either 30S or 70S ribosomes [4,52,60,61,62,63]. In the canonical pathway, formation of the 30S initiation complex is orchestrated by synergistic interactions of IF1, IF2, and IF3, which modulate mutual binding affinities to optimize tRNA^fMet^ recruitment to 30S [4,10]. Codon–anticodon recognition stabilizes the 30S IC, enabling subsequent 50S subunit joining [4,64], IF dissociation, and progression to the elongation-competent 70S IC [4,9,10,65,66,67,68,69,70,71]. Non-canonical 70S initiation complex formation pathways include leaderless mRNA initiation [62,63], translation re-initiation [72,73], polysome-associated initiation [74,75,76,77], and 70S scanning [61].

We progressively expanded the system toward a multicomponent complex using Bpy-tRNA^fMet^ as the reporter molecule. Elevated concentrations of Mg^2+^ stabilize the tertiary structure of rRNA, potentially facilitating binding of mRNA and tRNA^fMet^ to the 30S subunit [42,55,58,78]. Thus, we examined initiator tRNA^fMet^ binding to the 30S•mRNA bimolecular complex (Figure 4c) across a range of magnesium ion concentrations (Figure S3a). We observed robust ternary 30S•mRNA•Bpy-tRNA^fMet^ complex formation under increasing Mg^2+^ conditions (3.1–100 mM) (Figure S3a). Subsequent system expansion involved adding individual initiation factors to dissect their roles, with each titrated against the 30S•mRNA•Bpy-tRNA^fMet^ ternary complex to assess contributions to tRNA binding efficiency. Considering the role of magnesium ions in Bpy-tRNA^fMet^ binding to the ribosome (Figure S3a), we conducted experiments at 7 mM or 20 mM Mg^2+^ (Figure S3b). Thermophoretic shifts occurred only with IF2, reflecting its role in recruiting tRNA^fMet^ by adopting an extended conformation and enhancing tRNA association [59,77,78,79]. Additionally, we demonstrated the versatility of the system by analyzing 30S IC formation efficiency as a function of IF3 in the presence of mRNA lacking a canonical start codon and regulatory elements (polyU mRNA) (Figure S3c). 30S ICs were practically not formed in the presence of polyU mRNA, potentially reflecting both the absence of productive interaction and the proofreading function of IF3 during codon–anticodon checking.

**Figure 5:**
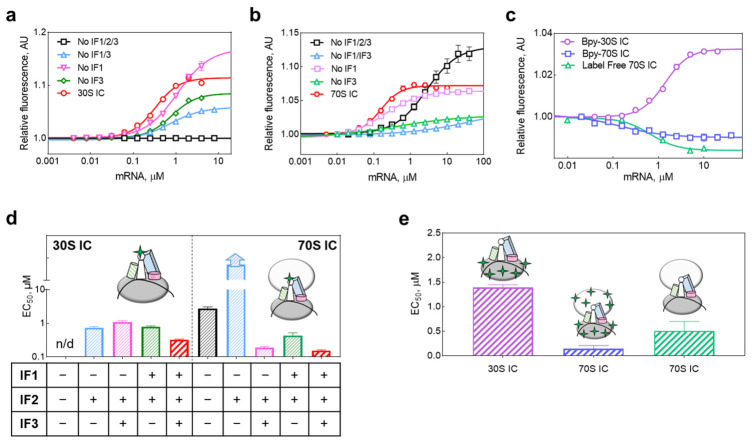
MST analysis of stepwise translation initiation complex assembly. 30S IC and 70S IC formation as a function of mRNA concentration, depending on the presence of initiation factors, using Bpy-tRNA^fMet^ (**a**,**b**) or (**c**) Bpy-30S/Bpy-70S as reporter ligands or label-free conditions. EC_50_ values for 30S IC and 70S IC formation using Bpy-tRNA^fMet^ (**d**), Bpy-30S/Bpy-70S ribosomes, or label-free conditions (**e**). Error bars indicate standard deviations from triplicate measurements. The upward arrow indicates lower bounds for *K*_D_ when the curve did not reach saturation (actual *K*_D_ is ≥the indicated value).

We next investigated 30S IC and 70S IC formation as a function of mRNA, using initiator Bpy-tRNA^fMet^ or Bpy-70S as reporter molecules or performing label-free measurements, while sequentially adding initiation factors to the system (Figure 5). mRNA titration revealed maximal efficiency of 30S IC formation when all three IFs were present (Figure 5a). The observed ≈2-fold drop in mRNA binding affinity and reduced thermophoresis in the absence of IF3 (or IF3 + IF1) support a stabilizing role for IF3 CTD–mRNA contacts in maintaining the SD:anti-SD placement and promoting accurate start-codon positioning before tRNA arrival [59]. The ≈3-fold drop in affinity upon IF1 removal, without a substantial change in signal amplitude, is consistent with IF1 providing key anchoring points for IF2 and IF3 that enhance their activity [59]. Thus, IF1 appears to reinforce factor-mediated mRNA engagement and fidelity without markedly altering the predominant conformational state detected by thermophoresis (Figure 5a,d). These findings demonstrate the synergistic contributions of IF1, IF2, and IF3 to 30S IC formation.

Binding of Bpy-tRNA^fMet^ to the 70S ribosome is characterized by a decrease in fluorescence signal with minimal change in signal amplitude (Figure 4c), corresponding to a *K*_D_ of 1 ± 0.1 μM (Figure 4d). Addition of mRNA to the 70S•Bpy-tRNA^fMet^ bimolecular complex reversed the thermophoretic direction and increased signal amplitude (Figure 5b,d), yielding a ternary 70S•mRNA•tRNA^fMet^ complex with an affinity of approximately 2.7 ± 0.3 μM. We observe formation of the ternary complex at excess mRNA concentrations (up to 40 μM), while Bpy-tRNA^fMet^ is present at sub-stoichiometric amounts (0.5 μM relative to 1 μM ribosomes), which may suggest a scaffolding or organizing role for mRNA in driving factor-independent 70S IC assembly. The high affinity of 70S for mRNA alone (*K*_D_ of 45 ± 4 nM) (Figure 4c,d) compared to tRNA suggests that mRNA binding is the primary event. Notably, in our experiments, we did not pre-dissociate 70S ribosomes into subunits before the assay; nevertheless, we observed efficient mRNA binding and ternary complex formation under these conditions, which may represent an expansion of our understanding of 70S-scanning initiation [61]. The opposite signal direction indicates that the 70S•mRNA•tRNA^fMet^ complex has altered thermophoretic properties (size, charge, or hydration shell) compared to the binary complexes, confirming genuine ternary complex formation rather than competitive binding. As observed for 70S IC, absence of any IFs decreased mRNA affinity relative to the complete system; exclusion of IF3 alone caused a >2-fold reduced affinity, while combined omission of IF3 and IF1 caused a >50-fold decreased affinity, accompanied by reduced thermophoretic amplitude. The absence of IF1 had minimal impact on 70S IC assembly efficiency. We further analyzed 70S IC formation efficiency as a function of mRNA concentration using Bpy-70S ribosomes, in the presence or absence of initiation factors (Figure S3d). The Bpy-70S•mRNA•tRNA^fMet^ ternary complex formed with comparable efficiency (3.1 ± 0.2 μM; Figure S3d) to that measured using Bpy-tRNA^fMet^ (2.7 ± 0.3 μM; Figure 5d). In the absence of IF1/IF3, mRNA binding to Bpy-70S•IF2•tRNA^fMet^ during 70S IC formation occurred with higher affinity (4.8 ± 0.9 μM; Figure S3d) than for complexes using Bpy-tRNA^fMet^ as the reporter molecule (Figure 5d).

We further used three complementary MST strategies to monitor 30S IC and 70S IC formation, employing labeled ribosomes or label-free configurations (Figure 5c,e). Ligand affinities during IC assembly were comparable across all approaches and aligned with values obtained using Bpy-tRNA^fMet^ as reporter (Figure 5d,e). The directionality of thermophoretic signals was identical for Bpy-tRNA^fMet^ and Bpy-30S during 30S IC formation (Figure 5a,c). For 70S IC assembly, Bpy-70S or unlabeled ligands produced descending MST traces with increasing mRNA concentration—opposite to the Bpy-tRNA^fMet^ signal (Figure 5c)—while IFs absence experiments (lacking IF1, IF3, or all IFs) with Bpy-70S yielded ascending traces as to Bpy-tRNA^fMet^ (Figure S3d). All the configurations delivered consistent EC_50_ values (0.3–1 μM).

### 2.4. MST Detection of Translation Inhibitors

Inhibitors of diverse chemical nature frequently target critical stages of translation, ultimately arresting or disordering protein synthesis [80,81,82,83]. These compounds bind to specific functional centers on the ribosome and disrupt key steps in codon recognition, peptide bond formation, and others. Such specificity minimizes impact on host eukaryotic ribosomes, making antibiotics a cornerstone therapy against bacterial infections [81,84,85,86,87]. Quantifying the binding affinities of antibiotics to their targets on the ribosome is an important parameter for developing novel therapeutics and combating emerging antibiotic resistance.

**Figure 6:**
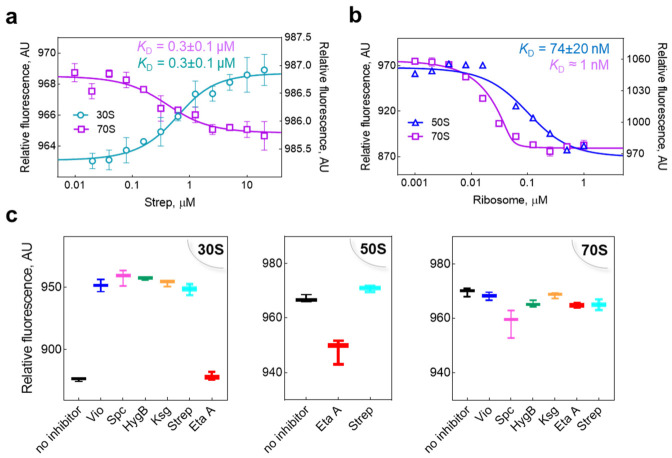
Bimolecular ribosome–inhibitor interactions by MST. (**a**) Streptomycin affinity for 30S/70S ribosomes: *K*_D_ = 0.3 ± 0.1 µM (both). (**b**) Bpy-erythromycin affinity for 50S/70S: *K*_D_ (Bpy-Ery•50S) = 74 ± 20 nM; *K*_D_ (Bpy-Ery•70S) ≈ 1 nM. (**c**) Thermophoresis shift in fluorescently labeled ribosomes in the presence of translation inhibitors: the antibiotics viomycin (Vio), spectinomycin (Spc), hygromycin B (HygB), streptomycin (Str), and etamycin A (EtaA).

Streptomycin (Str), an aminoglycoside antibiotic, binds protein S12 and 16S rRNA within the 30S subunit and induces translational errors [88,89,90,91]. Despite extensive studies of streptomycin’s mechanism of action, direct quantitative data on its affinity for the bacterial ribosome were previously absent. Dihydrostreptomycin (DHSM), the most stable reduced analog of streptomycin (with –CHO replaced by –CH_2_OH), similarly binds to 16S rRNA of the 30S subunit and induces translational misreading. Equilibrium binding studies with *E. coli* ribosomes at 25 °C and 10 mM Mg^2+^ revealed *K*_D_ values of 94 nM (70S) and 1 μM (30S), respectively [89,92,93]. Using MST with Bpy-labeled ribosomes, we measured streptomycin dissociation constants of 0.3 ± 0.1 μM for both 30S and 70S ribosomes in the presence of 7 mM Mg^2+^, showing comparable affinities. The thermophoretic signals were in opposite directions (Figure 6a). The obtained affinity values fell within the range reported for dihydrostreptomycin.

MST’s versatility enables the use of virtually any ligand as a fluorescent reporter. In our study, we validate this statement using macromolecules (ribosomes), medium-sized proteins (initiation factors), and small molecules (antibiotics) as reporter molecules. We employed BODIPY-labeled erythromycin (Bpy-Ery) as a reporter to probe macrolide binding with the ribosome (Figure 6b). Erythromycin reversibly binds the 50S exit tunnel through direct contacts with 23S rRNA and influences nascent peptide egress [87,94,95,96]. Obtained affinities (*K*_D_ = 74 ± 20 nM for Bpy-Ery•50S; ~1 nM for Bpy-Ery•70S) aligned closely with prior reports [97].

In pharmaceutical development and biotechnology, rapid screening of small molecules as potential inhibitors represents an indispensable strategy for discovering novel therapeutic compounds. Microscale thermophoresis enables high-throughput screening of ligand specificity with high accuracy, requiring minimal sample volumes and time. Therefore, we screened changes in the mobility of Bpy-labeled ribosomes resulting from their binding to known antibiotics targeting the 30S subunit (viomycin, spectinomycin, hygromycin B, kasugamycin, and streptomycin) [80,81,90,98,99,100,101,102,103,104] and the 50S subunit (etamycin A) [105]. Pronounced mobility shifts were observed with isolated 30S and 50S subunits in complexes with the analyzed inhibitors, whereas 70S complexes exhibited muted thermophoretic responses.

### 2.5. MST Assessment of Inhibitor Effects on Translation Initiation

Elucidating the action mechanisms of translation inhibitors is essential for developing novel antimicrobials and understanding bacterial resistance profiles. Moreover, comprehensively studied inhibitors serve as probes for exploring fundamental aspects of translation. Therefore, we examined the effects of antibiotics, DNA aptamers, and a proline-rich antimicrobial peptide on 70S initiation complex formation using diverse fluorescent reporters.

**Figure 7:**
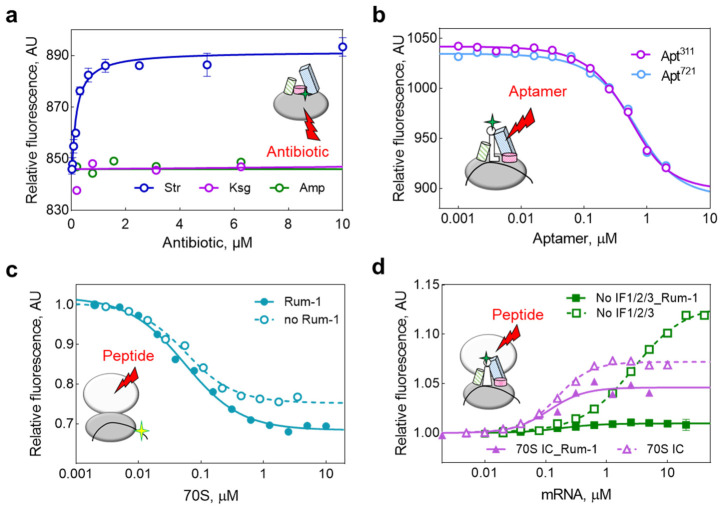
MST analysis of translation initiation complex formation in the presence of diverse inhibitors. (**a**) Monitoring the effects of A site binder streptomycin (Str), E site binder kasugamycin (Ksg), and translation-unrelated ampicillin (Amp) using IF1^Cy5^ on 30S•IFs. (**b**) Effects of IF2-targeting aptamers (Apt^311^, Apt^721^) on 30S IC formation, monitored using Bpy-tRNA^fMet^. (**c**) Binding curves of the bimolecular 70S•mRNA-Flu complex in the presence of rumicidin-1. (**d**) Efficiency of 70S IC formation in the presence of the proline-rich rumicidin-family peptide Rum-1. Analysis of 70S IC formation in the presence of Rum-1, dependent on initiation factors (IFs), was conducted via mRNA titration using Bpy-tRNA^fMet^ as the reporter ligand.

We first investigated streptomycin binding to the 30S•IFs complex by monitoring IF1^Cy5^, given that both agents target the ribosomal A site. Str titration produced upward MST traces, opposite to the 30S•IF1^Cy5^ binding signal (Figure 4a), that can be suggesting Str reduces IF1 affinity (Figure 7a). Dose–response fitting yielded an EC_50_ of 0.14 μM.

Another antibiotic targeting the 30S ribosome that we investigated was kasugamycin (Ksg). This aminoglycoside representative [106] binds to the 30S ribosomal subunit at the E site in the mRNA binding channel region between h24 and h28 of 16S rRNA, disrupting the transition from 30S PIC to 30S IC [107,108]. Kasugamycin is thought to sterically hinder mRNA positioning at the E site, thereby affecting initiator tRNA^fMet^ placement at the ribosomal P site. Meanwhile, Ksg shows no significant effect on mRNA, IF1, or IF3 binding to the 30S ribosome [108]. In our experiments, kasugamycin presence in the 30S•IFs complex, monitored via IF1^Cy5^, did not alter the thermophoresis signal, consistent with existing data showing no effect on IF1.

We also examined the effect of ampicillin (Amp) on 30S•IFs complex formation, monitored via IF1^Cy5^. This β-lactam antibiotic from the aminopenicillin class inhibits bacterial cell wall peptidoglycan synthesis by binding to penicillin-binding proteins and exerts no direct effect on translation [109,110]. In our experiments, the presence of Amp exerted no effect on 30S•IFs formation (Figure 7a).

Aptamers are chemically synthesized short oligonucleotides selected by SELEX (Systematic Evolution of Ligands by EXponential enrichment) that specifically bind target molecules [111,112]. We next employed the 30S IC assembly assay to evaluate IF-targeting DNA aptamers using Bpy-tRNA^fMet^—which binds IF2—as the reporter ligand (Figure 7b). Titration of Apt^311^ and Apt^721^, both specific for IF2, produced downward MST traces [113], yielding EC_50_ values of 0.3 and 0.4 μM, respectively, upon dose–response fitting.

Proline-rich antimicrobial peptides represent promising antibacterial candidates, exhibiting broad-spectrum activity against bacteria, viruses, fungi, and cancer cells, and offering an alternative to conventional drugs amid rising antibiotic resistance [114,115,116]. Given the established finding that Rum-1 occupies the 50S exit tunnel, sterically impeding P and A site tRNA accommodation at the peptidyl transferase center and thereby inhibiting peptide bond formation [117], we examined the effects of this proline-rich rumicidin-family peptide on 70S initiation complex formation efficiency (Figure 7c,d and Figure S4). Rum-1 did not perturb mRNA affinity in the 70S•mRNA bimolecular complex, though a modest thermophoretic amplitude increase suggested altered complex mobility without direct competition at the mRNA binding site (Figure 7c). However, Rum-1 enhanced mRNA affinity by 15.5-fold in the 70S•mRNA•Bpy-tRNA^fMet^ ternary complex, accompanied by a 15-fold reduction in MST amplitude. Addition of initiation factors partially counteracted this effect, modestly reducing mRNA affinity while diminishing MST amplitude 1.7-fold (Figure 7d and Figure S4).

## 3. Discussion

Translation initiation in prokaryotes constitutes a tightly regulated, multicomponent process that governs the efficiency and fidelity of protein synthesis (Figure 1). Biochemical study of this pathway faces significant challenges due to the complex system of cooperative and sequential interactions between the ribosome, mRNA, initiator tRNA^fMet^, and initiation factors, in addition to limitations of traditional approaches such as radiolabeling. Biophysical methods, including surface plasmon resonance (SPR) and isothermal titration calorimetry (ITC), are commonly employed to determine biomolecular affinities. We utilize a fluorescence-based analytical platform centered on microscale thermophoresis, which provides a robust, near-native environment for translation initiation studies (Figure 2). The MST-method offers key advantages over SPR and ITC, including no requirement for sample immobilization, lower sensitivity to buffer conditions, rapid experimental throughput, and minimal sample consumption. However, microscale thermophoresis requires one interaction partner to bear a fluorescent label, which can be limiting in cases where labeling is challenging or undesirable; at the same time, this requirement imparts flexibility by allowing selection of the reporter molecule, enabling analysis across multiple labeling strategies and configurations. Thermophoresis can be described as a directed particle flux proportional to the temperature gradient, which in steady state is balanced by ordinary diffusion:

$$j=-cD_{T}gradT,$$

where j—particle flux, c—molecular concentration, D_T_—thermodiffusion coefficient, and T—temperature; the resulting thermophoretic concentration change is defined by the Soret coefficient (S_T_ = D_T_/D, where D_T_ is the thermophoretic coefficient, and D is the diffusion coefficient [118]. For a given spatial temperature difference ΔT, the concentration change in the steady state is determined as

$$\frac{c_{hot}}{c_{cold}}=exp\left(-S_{T}∆T\right),$$

where c_hot_ is the molecular concentration in the heated region, and c_cold_ is the molecular concentration in the cold region. The Soret coefficient can be either positive or negative depending on the properties of the labeled molecule (size, charge, and solvation), resulting in a steady-state concentration in the heated zone that is either lower or higher than the initial concentration, and thus MST signals that increase or decrease with ligand concentration [118]. In multi-component systems such as bacterial translation initiation involving ribosomes, tRNA, and initiation factors, different fluorescently labeled components (for example, Bpy-Met-tRNA^fMet^ and Bpy-70S) exhibit fundamentally different thermophoretic properties due to their vastly different sizes (~25 kDa vs. ~2.5 MDa), surface charges, and hydration shells, which can result in opposite signal directions for the same binding event (Figure 5b,c). However, this variation in signal direction does not affect the accuracy of *K*_D_ determination, as both positive and negative thermophoresis yield equivalent dissociation constants. Since at least one of these parameters changes upon binding, MST enables the detection of a wide range of interactions. The technique quantifies binding through the change in normalized fluorescence, reflecting global changes in the fluorescently labeled species rather than localized effects. This is particularly advantageous for studying multi-component complexes such as the bacterial translation initiation system, where MST requires fluorescent labeling of only one component while all other proteins/ligands remain in their native state, enabling analysis of interdependencies between multiple interaction partners with minimal material consumption and without the stability issues associated with surface-coupled assays. The use of different reporter molecules in our work serves as internal validation, as consistent *K*_D_ values obtained with different labels confirm that the same interaction is being measured regardless of signal direction.

In our study, we performed a sequential analysis of molecular affinities of ligands during the formation of 30S and 70S initiation complexes, employing each of them as the fluorescent reporter in turn. We introduce a rapid BODIPY-labeling protocol for ribosomes and subunits (Figure 3a), with labeling efficiency and functional integrity—including formation of active 70S initiation complexes capable of peptide bond formation—confirmed in control tests (Figure S1a and Figure 3b–d). Maintaining conformational stability and resistance to aggregation in protein preparations is critical for preserving functional activity, particularly upon modifications; here, we comprehensively confirmed ribosomal integrity across extensive control tests—including, for the first time, nanoDSF application to ribosomes under varying ionic conditions—demonstrating no significant structural perturbations from BODIPY labeling (Figure 3e and Figure S1c; Table S1).

We initiated the analysis of translation initiation using our platform by examining bimolecular interactions between 30S/70S ribosomes and ligands serving as reporter molecules (IF1^Cy5^, IF2^Cy5^, IF3^Cy5^, mRNA-Flu, and Bpy-tRNA^fMet^). We observed high-affinity binding of initiation factors and mRNA to the 30S ribosome (Figure 4a–d). Initiator tRNA^fMet^ binding was notably less efficient, consistent with its dependence on initiation factors (Figure 4c,d).

Microscale thermophoresis offers high sensitivity and solution-phase flexibility for dissecting dynamic supramolecular assemblies across diverse biological systems [18,119,120]. We utilized the MST platform to investigate translation initiation as a multicomponent system using a diverse set of fluorescently labeled molecules. By progressively increasing system complexity, we demonstrated the role of magnesium ions in mRNA and tRNA^fMet^ binding to the 30S ribosome (Figure S3a,b). Quantitative analysis of 30S IC formation showed the individual and synergistic contributions of IF1, IF2, and IF3, confirming IF2’s dominant role in initiator tRNA^fMet^ recruitment [10,121] while revealing IF1/IF3-mediated modulation of mRNA and tRNA^fMet^ affinities (Figure 5 and Figure S3d). These equilibrium measurements complement kinetic insights from stopped-flow approaches and highlight the unique capacity of MST to capture cooperative binding under near-native conditions. We applied our model system to analyze the formation of both 30S and 70S initiation complexes, using labeled tRNA^fMet^ as an example of a reporter ligand, along with labeled ribosomes or a completely label-free setup in our experiments (Figure 5). Remarkably, all the approaches yielded consistent EC_50_ values (0.3–1.2 μM) despite execution across distinct project phases, instruments, locations, and operators. This underscores the system’s experimental robustness.

Translation is a frequent target for inhibitors of diverse origins. Accordingly, developing model systems that support not only the elucidation of fundamental mechanisms but also the assessment of potential inhibitors holds substantial importance—this approach enables both decoding their mechanisms of action for therapeutic development and addressing key questions in basic translation research. In addition to analyzing the affinity of interacting molecules, the model system we present can also be used for these purposes, namely, evaluating the effects of potential inhibitors on the translation initiation stage. Using microscale thermophoresis, we analyzed the effects of antibiotics, DNA aptamers, and an antimicrobial peptide on translation initiation. We showed the potential of our system as a platform for rapid screening of potential inhibitors’ binding efficiency to their target, exemplified by the interaction of known antibiotics (viomycin, spectinomycin, hygromycin B, kasugamycin, streptomycin, and etamycin A) with the ribosome (Figure 6c). As an example of targeted affinity analysis for small molecules such as the antibiotics streptomycin or erythromycin with the ribosome, we performed measurements under alternative experimental configurations in which the reporter molecule was either the target (ribosome) (Figure 6a) or the ligand (antibiotic) (Figure 6b). As a result, we determined that streptomycin binds the 70S ribosome and the 30S ribosomal subunit with similar affinity. Analysis of Bpy-erythromycin binding to the 70S ribosome and the 50S ribosomal subunit revealed high-affinity interactions, with dissociation constants of ≈1 nM and 74 ± 20 nM, respectively, in agreement with previously reported data [97].

In addition to analyzing bimolecular interactions, we used our model system to assess the effects of inhibitors on translation initiation within a multicomponent complex. We showed that Rum-1 also affects the formation of the 70S initiation complex, in which the presence of initiation factors plays an important role in this process. These findings open a new direction for investigating how potential antimicrobial inhibitors act on the translation process. We also demonstrated the use of this model system to analyze the effects of antibiotics, using streptomycin and kasugamycin as examples, as well as IF2-targeting DNA aptamers, on the ribosomal translation initiation complex (Figure 7a,b).

The model system we present, based on the MST platform, provides a sensitive biophysical toolkit for dissecting bacterial translation initiation by minimizing sample consumption while preserving sensitivity and physiological relevance, thereby enabling comprehensive characterization of initiation complex intermediates and surpassing fluorescence methods that require extensive manipulation or specialized instrumentation. Its versatility may extend beyond bacterial systems to eukaryotic initiation complexes, ribosome biogenesis, elongation/termination, and other supramolecular assemblies like spliceosomes, expressome complexes, RNPs, and transcription machineries, addressing core questions in complex assembly, regulation, and stability across organisms; nanoDSF integration further enables monitoring of protein modifications, while rapid affinity screening—including for limited samples—supports drug development.

## 4. Materials and Methods

### 4.1. Biological Preparations

#### 4.1.1. 70S Ribosomes and Ribosomal Subunits

The 70S ribosomes and 30S/50S subunits were prepared as described previously [3]. Subunits were isolated from purified 70S ribosomes by sucrose gradient centrifugation (Ti-15 rotor, Beckman Coulter, Brea, CA, USA) under dissociating conditions in buffer TAKM_1.25_ (50 mM Tris-HCl, pH 7.5, 70 mM NH_4_Cl, 30 mM KCl, 1.25 mM MgCl_2_). Ribosome concentration was determined by measuring the absorbance at 260 nm, where 1 unit corresponds to 23 pmol of 70S, 63 pmol of 30S, and 37 pmol of 50S ribosomes, respectively.

#### 4.1.2. Translation Factors Purification

Initiation factors IF1, IF2, and IF3 were purified as described previously [15]. Elongation factors EF-Tu and EF-G were isolated following established protocols [122]. Mutant variants engineered for site-specific labeling—IF1 D5C, IF2γ G810C, and IF3 E166C—were expressed and purified according to published procedures [3,122,123].

### 4.2. Fluorescence Ligands

#### 4.2.1. BODIPY-Met-tRNA^fMet^ (Bpy-tRNA^fMet^)

The fluorescent BODIPY FL SSE dye (D6140, Invitrogen, San-Diego, CA, USA) was used to generate fluorescently labeled initiator Met-tRNA^fMet^, following a labeling procedure analogous to that described previously [15].

#### 4.2.2. Fluorescein-Labeled mRNA (mRNA-Flu)

Synthetic mRNA mMF1 (5′-ACUAUGUUU-3′) [15] was produced by in vitro transcription for 3 h at 37 °C. Reactions contained transcription buffer (40 mM Tris-HCl, pH 7.5, 15 mM MgCl_2_, 2 mM spermidine, 10 mM NaCl), 10 mM DTT, 2.5 mM NTPs, 5 mM GMP, 0.01 U/ µL inorganic pyrophosphatase, 2 U/ µL T7 RNA polymerase, and 5 ng/ µL DNA template. Transcripts were purified by three sequential ethanol precipitations followed by anion-exchange chromatography on a HiTrap Q column (5 mL; 17-1151-01, GE Healthcare, Chicago, IL, USA), equilibrated with buffer A (50 mM Tris-HCl, pH 7.0, 5 mM EDTA, 0.3 M NaCl). mRNA was eluted by a linear gradient of 0.3–1.5 M NaCl in buffer A, visualized by 8 M urea-PAGE and Methylene blue staining, pooled, and recovered by three additional ethanol precipitations. mRNA was fluorescently labeled with fluorescein (M041030, Sigma-Aldrich, St. Louis, MO, USA) as described previously [3].

#### 4.2.3. Fluorescent Labeling of IFs (IF1^Cy5^, IF2^Cy5^, IF3^Cy5^)

Mutant IFs (pre-incubated with 300 µM TCEP for 10 min at 37 °C) were mixed with maleimide-Cy5 (GE Healthcare Life Sciences, Chicago, IL, USA) at a 10:1 molar ratio in labeling buffer (25 mM Tris-HCl, pH 7.1, 100 mM NH_4_Cl, 10% glycerol). Reactions proceeded for 2 h at room temperature in the dark with gentle mixing every 30 min, then were quenched with 6 mM β-mercaptoethanol. Labeled proteins were purified by cation-exchange chromatography on HiTrap SP HP columns (GE Healthcare Life Sciences, Chicago, IL, USA). IF1 and IF3 were eluted by a gradient of 50–1000 mM NH_4_Cl; IF2 by a gradient of 30–750 mM NaCl. Purified proteins were dialyzed against storage buffer (25 mM Tris-HCl, pH 7.1, 200 mM NH_4_Cl, 10% glycerol, 6 mM β-mercaptoethanol), aliquoted, and stored at −80 °C. Fractions were analyzed by SDS-PAGE (15% for IF1/IF3; 10% for IF2) with Coomassie brilliant blue staining. Protein concentrations and degree of labeling were determined using the Cy5 extinction coefficient (ε = 250,000 M^−1^ cm^−1^).

#### 4.2.4. BODIPY-Labeled Ribosomes (Bpy-70S, Bpy-50S, Bpy-30S)

Ribosomes were fluorescently labeled at free surface amino groups of ribosomal proteins using NHS-ester BODIPY FL SSE dye (Bpy, D6140, Invitrogen, San-Diego, CA, USA). The 70S ribosomes (5 µM) were incubated with Bpy (1:10 molar ratio) in HAKM_7_ buffer (50 mM HEPES, pH 8.7, 70 mM NH_4_Cl, 30 mM KCl, 7 mM MgCl_2_) for 1 h at 25 °C in the dark. Unbound dye was removed by centrifugation through a 1.1 M sucrose cushion in TAKM_7_ buffer (50 mM Tris-HCl, pH 7.5, 70 mM NH_4_Cl, 30 mM KCl, 7 mM MgCl_2_) at 259,000× *g* for 2 h at 4 °C (TLS-55 rotor, Beckman Coulter, Brea, CA, USA). Pellets were resuspended in TAKM_7_.

Fluorescently labeled 30S and 50S subunits were obtained by dissociating Bpy-70S in TAKM_1.25_ buffer (50 mM Tris-HCl, pH 7.5, 70 mM NH_4_Cl, 30 mM KCl, 1.25 mM MgCl_2_) for ≥1 h at 4 °C, followed by separation through a 10–40% sucrose gradient at 28,000 rpm for 19 h at 4 °C (SW28 rotor, Beckman Coulter, Brea, CA, USA) (Figure 3a). Gradients were fractionated based on absorbance profiles at 260 nm, and the fractions containing ribosomal subunits were collected and pelleted by centrifugation at 50,000 rpm for 18 h at 4 °C (SW55 rotor, Beckman Coulter, Brea, CA, USA). Labeling efficiency was verified by 10% SDS-PAGE.

### 4.3. Biochemical Reactions

#### 4.3.1. MST Experiments

MST samples were prepared on ice in the dark with fixed fluorescent reporter concentrations (Bpy-tRNA^fMet^, mRNA-Flu, IFs^Cy5^, Bpy-70S, Bpy-30S) and serial 1:1 dilutions of titrants, mixed by gentle pipetting. Mixtures were incubated for 30 min at 37 °C in TAKM_7_ (50 mM Tris-HCl, pH 7.5, 70 mM NH_4_Cl, 30 mM KCl, 7 mM MgCl_2_) or TAKM_20_ (50 mM Tris-HCl, pH 7.5, 70 mM NH_4_Cl, 30 mM KCl, 20 mM MgCl_2_), centrifuged (13,000 rpm, 5 min, 25 °C) and loaded into standard treated capillaries (MO-K022) or label-free capillaries (MO-Z022) (NanoTemper Technologies GmbH, Munich, Germany).

Samples containing Bpy or Flu fluorophores were measured on a Monolith NT.115 instrument (green LED, 40% IR laser, NanoTemper Technologies GmbH, Munich, Germany). Samples containing Cy5-labeled components were measured on a Monolith NT.LabelFree instrument (red LED, 10% IR laser, NanoTemper Technologies GmbH, Munich, Germany). Label-free measurements employed 10% IR laser. The 30S subunits were activated for 1 h at 37 °C in TAKM_20_ prior to assays.

The use of diverse fluorescent reporters throughout this study enabled analysis of binding interactions in different detection channels (green and red LEDs) and in the absence of fluorescence labels (label-free mode). This versatility allowed cross-validation of results across multiple detection filters and minimized potential artifacts arising from a single reporter type. The consistency of ligand binding affinities obtained with different reporters confirmed that the observed thermophoretic changes reflected genuine binding events rather than reporter-specific effects. This multi-reporter approach strengthens the reliability of our conclusions and enables direct comparison of results across experiments.

##### Bimolecular Binding

Binding affinities of Cy5-labeled IFs to 30S subunits were measured at fixed IF concentrations (0.05 µM IF1^Cy5^/0.15 µM IF2^Cy5^/0.15 µM IF3^Cy5^) with varying 30S (7.8 nM–2 µM) concentrations. mRNA binding to ribosomes was assessed using 0.025 µM mRNA-Flu reporter with 30S (3.9 nM–2 µM) or 70S (2 nM–3.5 µM) ribosomes. Initiator tRNA^fMet^ binding employed 0.5 µM Bpy-tRNA^fMet^ with 30S (1 nM–6 µM) or 70S (5 nM–10 µM). All complexes were incubated for 30 min at 37 °C.

The affinity of streptomycin was analyzed using 0.5 µM Bpy-30S or 0.2 µM Bpy-70S with antibiotic concentrations from 9.8 nM to 20 µM. Erythromycin binding employed varying ribosome concentrations (0.1 nM–1 µM) at fixed 0.05 µM Bpy-Ery. Diffusion shifts in the inhibitors were assessed with 0.5 µM Bpy-30S, 0.5 µM Bpy-50S, and 0.2 µM Bpy-70S in the presence of 200 µM of each antibiotic: viomycin (Vio), spectinomycin (Spc), hygromycin B (Hyg B), kasugamycin (Ksg), streptomycin (Str), and etamycin A (Eta A).

##### Multicomponent System

The effect of Mg^2+^ on mRNA and tRNA^fMet^ binding to 30S ribosomes was analyzed using 0.5 µM Bpy-tRNA^fMet^, 4 µM mRNA, 1 µM 30S, and Mg^2+^ concentrations from 3.1 to 100 mM. IF-dependent 30S initiation complex formation was assessed with 1 µM 30S, 0.5 µM Bpy-tRNA^fMet^, 4 µM mRNA, 0.5 mM GTP, and 0.3–10 µM IFi in TAKM_7_ and TAKM_20_ buffers.

The 30S/70S initiation complex formation was analyzed in the presence of initiation factors using 0.5 µM Bpy-tRNA^fMet^, 1 µM 30S/70S, 2 µM IF1, 1 µM IF2, 1.5 µM IF3, 0.5 mM GTP, and mRNA (4 nM–8 µM for 30S IC; 5 nM–40 µM for 70S IC), with incubation at 37 °C for 30 min. Ribosome-monitored assays employed 0.5 µM Bpy-30S and 0.2 µM Bpy-70S; label-free setups used 1 µM 70S. 70S IC formation with Bpy-70S followed identical ligand conditions. 30S IC formation as a function of IF3 (1 nM–2.5 µM) was assessed with mRNA-Flu or polyU mRNA (UUU-UUU-UUU-…) under the same conditions.

##### Inhibitors Action Analysis

The effects of 40 µM streptomycin, 100 µM kasugamycin, and 100 µM ampicillin on initiation factor binding to 30S ribosomes were analyzed by monitoring thermodiffusion changes of 0.05 µM IF1^Cy5^, with complex ligands at 0.05 µM 30S, 0.15 µM IF2, and 0.15 µM IF3 in TAKM_7_ buffer. IF2-targeted aptamer effects on 30S IC formation were assessed using 0.5 µM Bpy-tRNA^fMet^, 1 µM 30S, 2 µM IF1, 1 µM IF2, 1.5 µM IF3, 0.5 mM GTP, and 4 µM mRNA. The aptamers (Apt^311^, Apt^721^) were activated by heating at 95 °C for 5 min, followed by 30 min incubation at room temperature, and tested across 0.1 nM–2 µM concentrations.

The effect of rumicidin-1 on 70S•mRNA (mRNA-Flu) and 70S IC ± IFs (Bpy-tRNA^fMet^) complex formation was assessed using a twofold serial dilution starting from 20 µM. An experiment on the effect of Rum-1 on the formation of the bimolecular 70S•mRNA-Flu complex was performed in the presence of 2 µM peptide. The efficiency of 70S IC formation as a function of mRNA in the presence of Rum-1 was assessed analogously to the experiments on initiation complex formation efficiency described above. The concentrations of the ligands were 0.5 µM Bpy-tRNA^fMet^, 1 µM 70S, 2 µM IF1, 1 µM IF2, 1.5 µM IF3, 0.5 mM GTP, 4 µM mRNA, and 2 µM rumicidin-1.

#### 4.3.2. nanoDSF Experiments

Conformational integrity and aggregation stability of fluorescently labeled Bpy-70S, Bpy-50S, and Bpy-30S were monitored by nanoDSF using a Prometheus NT.48 (NanoTemper Technologies GmbH, Munich, Germany) in TAKxMy buffer (50 mM Tris-HCl, pH 7.5, 70 mM NH_4_Cl, X mM KCl, Y mM MgCl_2_). K^+^ concentration was either 30 or 360 mM; Mg^2+^ was used at 0, 1, 5, 7, 20, or 50 mM concentration.

The samples (1 µM 70S/50S/30S/Bpy-70S/Bpy-50S/Bpy-30S in a 10 µL volume) were loaded into the glass capillaries (PR-C002, NanoTemper Technologies GmbH, Munich, Germany). Optimal LED intensity (10–30%) was determined by initial fluorescence scan, followed by heating in the range of 20–95 °C at 1 °C/min. Melting points (Tm) and aggregation onset temperatures were calculated using PR.ThermControl software v2.0.2 (NanoTemper Technologies GmbH, Munich, Germany).

#### 4.3.3. Kinetic Experiments

##### Rapid Kinetics

The 70S association and dissociation were monitored by stopped-flow light scattering (λ_ex_ = 430 nm) on an SX-20 system (Applied Photophysics, Leatherhead, UK) using native or fluorescently labeled ribosomes and IF3. Association reactions employed final concentrations of 0.15 µM Bpy-30S and 0.05 µM Bpy-50S. Dissociation of 0.1 µM 70S/Bpy-70S was initiated upon addition of 0.3 µM IF3. All the reactions were performed in TAKM_7_ buffer at 25 °C.

##### Slow Kinetics

The 30S IC formation was analyzed using 0.5 µM Bpy-30S (30S was reactivated as for MST experiments), 0.75 µM each of IF1, IF2, IF3, 0.75 µM fMet-tRNA^fMet^, 1 mM GTP, 1 mM DTT, and 2 µM mRNA in TAKM_7_ buffer. 70S IC formation was employed using 0.5 µM Bpy-70S/Bpy-30S + Bpy-50S mixtures with the same IFs, fMet-tRNA^fMet^, GTP, DTT, and mRNA concentrations in TAKM_7_. The mixtures were incubated for 60 min at 37 °C, and fluorescence emission was monitored at 512 nm. The efficiency of dipeptide bond formation in the resulting complexes was assessed according to the peptide synthesis protocol.

#### 4.3.4. Peptide Bond Formation

Peptide bond formation efficiency was assessed using native and fluorescently labeled 70S, 30S, and 50S ribosomes. The 70S ICs were formed by incubating 0.4 µM ribosomes, 1.6 µM mRNA (AUG-GUU-UUC) [16], 0.6 µM each IF1, IF2, IF3, and fMet-tRNA^fMet^ with 1 mM GTP and 1 mM DTT for 1 h at 37 °C. The 30S subunits were activated as described above. Ternary complexes (0.8 µM [^14^C]Val-tRNA^Val^•EF-Tu•GTP) were prepared by incubating 1.6 µM EF-Tu with 3 mM PEP, 1 mM GTP, and pyruvate kinase (1/100 vol) for 15 min at 37 °C, followed by the addition of 0.8 µM [^14^C]Val-tRNA^Val^ (2:1 EF-Tu:tRNA ratio) for 5 min at 37 °C.

Pre-translocation complexes were formed by mixing 70S ICs with the ternary complexes for 5 min at 37 °C. Dipeptide fMet-[^14^C]Val hydrolysis from peptidyl-tRNA was induced with 0.5 M KOH (1/10 vol) for 30 min at 37 °C and neutralized with glacial acetic acid (1/10 vol). Peptide bond formation efficiency was quantified by reversed-phase HPLC (RP-C8 column, Waters, Milford, MA, USA) using a 0–65% CH_3_CN gradient in 0.1% TFA, measuring radioactive label incorporation as described previously [124].

### 4.4. Data Analysis

Relative fluorescence was calculated using MO.Control software v1.6.1 (NanoTemper Technologies GmbH) from the F_hot_ and F_cold_ regions of MST traces. Binding affinities (*K*_D_) were determined for bimolecular interactions (Figure 4 and Figure 6), while EC_50_ was determined for larger multi-component complexes (Figure 5 and Figure 7), by fitting thermophoresis data as a function of ligand concentration using GraphPad Prism v.8.0.1 (GraphPad, San Diego, CA, USA). All data points represent the mean of 3–4 independent experimental replicates, with error bars showing standard deviation. The equilibrium dissociation constants (*K*_D_) were obtained by fitting a quadratic binding equation (Y = Y_Min_ + (Y_Max_ − Y_Min_)/2 × ([Z + X + *K*_D_] − [sqrt(((Z + X + *K*_D_)^2^) − (4ZX))])), where Z is the reporter concentration and X is the total ligand concentration. This full quadratic dependency based on the law of mass action allows precise determination of *K*_D_ even at reporter concentrations slightly higher than *K*_D_, since both the inflection point and the shape of the dose–response curve are taken into account. EC_50_ values were obtained by nonlinear regression using the equation (Y = Y_Min_ + (Y_Max_ − Y_Min_)/(1 + (EC_50_/X) n), where n is the Hill slope. For all reported *K*_D_/EC_50_ values, 95% confidence intervals were calculated, and the goodness of fit (R^2^) was ≥0.98. For experiments with relatively small signal changes, the following R^2^ values were obtained: Figure 4a: 0.82 for IF1^Cy5^•30S, 0.84 for IF2^Cy5^•30S, 0.74 for IF3^Cy5^•30S; Figure 4c: 0.86 for Bpy-Met-tRNA^fMet^•30S; Figure 5c: 0.90 for Bpy-70S IC, 0.96 for LabelFree experiment; Figure 6a: 0.91 for Bpy-30S•Strep, 0.86 for Bpy-70S•Strep; Figure 6b: 0.94 for Bpy-Ery•50S; Figure 7d: 0.91 for 70S IC with Rum1. The limitations of these parameters include EC_50_ values representing potency rather than true *K*_D_ when interactions are non-1:1 or show cooperativity (Hill slope ≠ 1); *K*_D_ determination may be less accurate when reporter concentration significantly exceeds *K_D_*, although the quadratic binding equation partially corrects for this. All graphs show individual data points with the corresponding nonlinear regression fits and 95% confidence bands.

## Data Availability

The original contributions presented in this study are included in the article/Supplementary Materials and pre-print in BIORXIV/2026/719552. Further inquiries can be directed to the corresponding author.

## Supplementary Materials

The following supporting information can be downloaded at: https://www.mdpi.com/article/10.3390/ijms27114953/s1, Refs. [125,126,127,128,129,130,131,132] are cited in this part.
